# Economic Evaluations of Digital Health Interventions for the Management of Musculoskeletal Disorders: Systematic Review and Meta-Analysis

**DOI:** 10.2196/41113

**Published:** 2023-07-06

**Authors:** Francis Fatoye, Tadesse Gebrye, Chidozie Mbada, Ushotanefe Useh

**Affiliations:** 1 Department of Health Professions Manchester Metropolitan University Manchester United Kingdom; 2 Lifestyle Diseases Research Entity North West University Potchefstroom South Africa

**Keywords:** musculoskeletal disorders, digital health interventions, cost-effectiveness, systematic review, digital health, intervention, management, musculoskeletal, muscles, joints, nerves, blood, pain, knee, hip

## Abstract

**Background:**

Musculoskeletal disorders (MSDs) are widespread in many countries and their huge burden on the society has necessitated innovative approaches such as digital health interventions. However, no study has evaluated the findings of cost-effectiveness of these interventions.

**Objective:**

This study aims to synthesize the cost-effectiveness of digital health interventions for people with MSDs.

**Methods:**

Electronic databases including MEDLINE, AMED, CIHAHL, PsycINFO, Scopus, Web of Science, and Centre for Review and Dissemination were searched for cost-effectiveness of digital health published between inception and June 2022 following the PRISMA (Preferred Reporting Items for Systematic Reviews and Meta-Analyses) guidelines. References of all retrieved articles were checked for relevant studies. Quality appraisal of the included studies was performed using the Quality of Health Economic Studies (QHES) instrument. Results were presented using a narrative synthesis and random effects meta-analysis.

**Results:**

A total of 10 studies from 6 countries met the inclusion criteria. Using the QHES instrument, we found that the mean score of the overall quality of the included studies was 82.5. Included studies were on nonspecific chronic low back pain (n=4), chronic pain (n=2), knee and hip osteoarthritis (n=3), and fibromyalgia (n=1). The economic perspectives adopted in the included studies were societal (n=4), societal and health care (n=3), and health care (n=3). Of the 10 included studies, 5 (50%) used quality-adjusted life-years as the outcome measures. Except 1 study, all the included studies reported that digital health interventions were cost-effective compared with the control group. In a random effects meta-analysis (n=2), the pooled disability and quality-adjusted life-years were –0.176 (95% CI –0.317 to –0.035; *P*=.01) and 3.855 (95% CI 2.023 to 5.687; *P*<.001), respectively. The meta-analysis (n=2) for the costs was in favor of the digital health intervention compared with control: US $417.52 (95% CI –522.01 to –313.03).

**Conclusions:**

Studies indicate that digital health interventions are cost-effective for people with MSDs. Our findings suggest that digital health intervention could help improve access to treatment for patients with MSDs and as a result improve their health outcomes. Clinicians and policy makers should consider the use of these interventions for patients with MSDs.

**Trial Registration:**

PROSPERO CRD42021253221; https://www.crd.york.ac.uk/prospero/display_record.php?RecordID=253221

## Introduction

Musculoskeletal disorders (MSDs), which comprise a wide range of inflammatory and degenerative conditions affecting the muscles, ligaments, joints, tendons, peripheral nerves, and supporting blood vessels, are widespread in many countries [1]. The burden of MSDs is substantial and increasing globally, particularly in the most economically deprived countries [2]. According to The Global Burden of Disease Study 2017 [3], the point prevalence and associated death rates of MSDs were 4151.1 and 1.0 per 100,000 population, respectively. According to Safiri et al [3], the prevalence estimate of MSDs was higher among females and increased with age; further, MSDs led to about 30.8 million disability-adjusted life-years worldwide, which is an increase of 3.4% from 1990 to 2017. Low back pain (LBP) is the main contributor to the overall burden of MSDs, but other contributors are rheumatoid arthritis (14 million), amputations (175 million), neck pain (222 million), osteoarthritis (343 million), and fractures (436 million) [4]. The cost of managing MSDs in the United States was estimated to be US $213 billion in direct and indirect costs, that is, 1.4% of the US gross domestic product, in 2011 [5]. It also cost the United Kingdom economy £10.2 billion (US $12.62 billion) in direct costs to the National Health System in 2017 [6].

In response to the growing burden of MSDs, the World Health Organization launched “the Rehabilitation 2030 Initiative” in 2017 [7]. This initiative draws attention to the importance of strengthening health system to provide rehabilitation, which is defined as a set of intervention designed to optimize functioning and reduce disability in individuals with health conditions in interaction with their environment. One of the innovative approaches to deliver rehabilitation to patients with MSDs is through the use of digital technologies. Digital health interventions may help overcome barriers to both delivering health care and care for chronic conditions [8]. Digital health interventions may include any intervention accessed through a computer, a smartphone, or any other hand-held device. Thus, digital health interventions have the advantage of being user flexible, where patients could receive care remotely without having to travel [9].

Compared with traditional models of care, digital health interventions are reported to have added benefits in providing evidence-based first-line care, low cost, and scalable patient education delivered via apps or web platform across a wide range of conditions [10,11]. Digital health intervention has also demonstrated its clinical benefit in the management of patients with MSDs [12]. To identify the most effective intervention with the least expenses, it is necessary to compare the costs and effectiveness of various digital health interventions. Apparently, there are no reviews that appraise individual studies and summarize results of the cost-effectiveness of digital health interventions on patients with MSDs. Therefore, this study was aimed to analyze evidence to identify whether digital health interventions are a cost-effective option for patients with MSDs.

## Methods

### Search Protocol and Registration

In this study, we used the PRISMA (Preferred Reporting Items for Systematic Reviews and Meta-Analyses) guideline (Multimedia Appendix 1), an evidence-based approach developed by Liberati in 2009 [13]. The systematic review protocol was also registered on PROSPERO (CRD42021253221).

### Search Strategy

We performed a systematic search of the following electronic databases to identify studies evaluating the cost-effectiveness of digital health interventions: MEDLINE, AMED, CIHAHL, PsycINFO, Scopus, Web of Science, and Centre for Review and Dissemination. The search was conducted from inception to June 2022. The included search terms are listed in Multimedia Appendix 2. Further, a manual search of reference sections of the included studies was performed to identify additional studies. The search was delimited to articles published in English.

### Inclusion and Exclusion Criteria

The inclusion criteria were economic evaluations (cost-effectiveness, cost-utility, cost-consequence, and cost-benefit analysis) performed in an adult population (aged >18 years) with musculoskeletal conditions; any form of digital health intervention delivered through an app or internet, with the comparators being no intervention, standard care, or any other nondigital forms of health interventions, was considered. We defined digital health interventions to include targeted client communication; personal health tracking; and on-demand information services delivered by apps, web-based software, or websites. The incremental cost-utility ratio (ICUR; ie, cost per quality-adjusted life-year [QALY]) and incremental cost-effectiveness ratio (ICER; ie, clinically relevant outcome such as pain intensity, disability, global perceived effect/recovery) were outcomes of the studies. The exclusion criteria were nonmusculoskeletal pathology, nondigital health interventions, study protocols, case studies/discussion papers, pilot studies, conference abstracts, and studies where the ICER was not calculated.

### Study Selection and Assessment of Methodological Quality

Following the removal of duplicates, titles and abstracts were screened by 2 reviewers (TG and FF) independently to identify eligible studies. The full texts of the identified studies were checked against the inclusion and exclusion criteria. The quality of the included studies was assessed and graded using the Quality of Health Economic Studies (QHES) instrument [14]. The QHES instrument consists of 16 items with scores ranging from 1 to 9, and the total score of the instrument is 100. During the assessment process, if the included study satisfied the criterion of an item, the study received an item-specific score; otherwise it received a score of 0. Each question has a weighted point value ranging from 1 to 9, which is used to generate a summary score from 0 to 100. As no standardized interpretation of the QHES exists, we adopted the score of 75-90 as good quality and a score of 90 and above as excellent quality, as applied by Tran and colleagues [15]. Two reviewers (TG and FF) participated in the study selection and methodological quality assessment. Any disagreement was resolved by discussion and consensus with the third reviewer (CM).

### Data Extraction

The following data were extracted by 2 reviewers (TG and FF): general information (author, year, and country), characteristics of participants (age, sample size, and disease type), description of intervention (duration of follow-up, intervention modalities [intensity and frequency], and comparator), economic evaluation (perspective, currency, types, cost/willingness to pay [WTP], and reference year), and study results (including clinical outcomes, costs, differences in clinical and costs outcomes, and ICER/ICUR). A summary table was used to display the extracted data. Disagreements were addressed through consultation with the third reviewer (CM).

### Data Analysis

The descriptive characteristics, quality of the included studies, and ICER/ICUR were descriptively analyzed. The quantitative results were carefully assessed for their suitability for meta-analysis. The included studies were grouped according to the disease type, time of follow-up, and clinical outcome measures. The mean costs and effects differences were then combined in a meta-analysis. A random effects meta-analysis model was used to pool the results from the individual studies. Because of the statistical evidence of heterogeneity across the included studies, a random effects model was chosen [16]. The assessment of heterogeneity was based on the *I*^2^ statistic. If substantial heterogeneity was present (ie, *I*^2^>50%), we tried to find an explanation for this heterogeneity.

All costs were converted to US dollars using purchasing power parities [17]. We adjusted the cost data to the reference year (2021) using the consumer price index from the World Bank website [18]. The intervention was considered cost-effective and not cost-effective when it was more effective and less costly and less effective and more costly compared with a comparator treatment, respectively [19].

### Ethical Approval

For this study ethical approval is not required.

### Informed Consent

The patient’s written informed consent was not made, as this was a systematic review study.

## Results

### Study Selection

**Figure 1:**
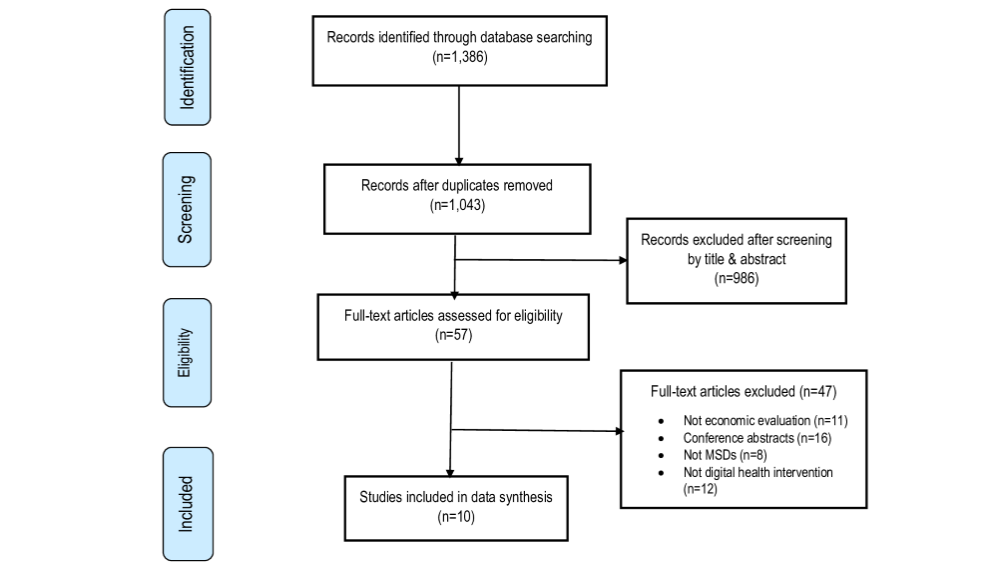
Flow diagram of publications included and excluded in the review.

We identified 1386 records in Scopus (n=1076); Web of Science (n=235); Centre for Review and Dissemination/NHS Economic Evaluation Database (n=13); and MEDLINE, AMED, CINAHL, PsycINFO (n=62; Figure 1). Of these records, 343 were duplicates. Following screening by titles and abstracts, 986 studies were excluded, leaving 57 articles for full-text review. After reading the full text of these articles, only 10 met the inclusion criteria and were eligible for this systematic review and meta-analysis.

### Quality of the Included Studies

The average QHES score for the included studies was 82.5. The detailed results of the quality assessment are shown in Table 1. Most of the included studies were conducted reasonably with good quality. However, many studies have failed to deal with subgroup analysis, discussion of potential bias, and use of best available sources.

**Table 1:** Quality assessment for the included studies.

Items	Suman et al [20]	Dear et al [21]	Zachwieja et al [22]	Fatoye et al [23]	Paganini et al [24]	Williams et al [25]	Hedman-Lagerlöf et al [26]	O’Brien et al [27]	Weinberger et al [28]	de Boer et al [29]
Was the study objective presented in a clear, specific, and measurable manner?	1^a^	1	1	1	1	1	1	1	1	1
Were the perspectives of the analysis (eg, societal, third-party payer) and reasons for its selection stated?	0.5^b^	1	0^c^	1	1	1	1	1	1	0
Were variable estimates used in the analysis from the best available source (ie, randomized control trial—best, expert opinion—worst)?	1	1	0	1	1	1	1	1	1	1
If estimates came from a subgroup analysis, were the groups prespecified at the beginning of the study?	0	0	1	0	0	0	0	0	0	0
Was uncertainty handled by (1) statistical analysis to address random events and (2) sensitivity analysis to cover a range of assumptions?	1	1	0	0	1	1	1	1	0	0
Was an incremental analysis performed between alternatives for resources and costs?	1	1	1	1	1	1	1	1	1	1
Was the methodology for data abstraction (including the value of health states and other benefits) stated?	1	1	1	1	1	1	1	Y	1	1
Did the analytic horizon allow time for all relevant and important outcomes? Were benefits and costs that went beyond 1 year discounted (3%-5%) and justification given for the discount rate?	1	1	1	1	1	1	1	1	1	1
Was the measurement of costs appropriate and the methodology for the estimation of quantities and unit costs clearly described?	1	1	1	1	1	1	1	1	1	1
Was the primary outcome measure(s) for the economic evaluation clearly stated and was the major short-term justification given for the measures/scales used?	0	0	0	0	0	0	0	0	1	1
Were the health outcomes measures/scales valid and reliable? If previously tested valid and reliable measures were not available, was justification given for the measures/scales used?	1	1	1	1	1	1	1	1	1	1
Were the economic model (including structure), study methods and analysis, and the components of the numerator and denominator displayed in a clear and transparent manner?	1	1	0	1	1	1	1	1	0	1
Were the choice of economic model, main assumptions, and limitations of the study stated and justified?	1	0.5	0.5	0.5	0.5	1	1	1	0.5	1
Did the author(s) explicitly discuss direction and magnitude of potential biases?	1	1	1	0	0	1	1	1	0	1
Were the conclusions/recommendations of the study justified and based on the study results?	1	1	1	1	1	1	1	1	1	1
Was there a statement disclosing the source of funding for the study?	1	1	0	1	1	1	1	1	0	1

^a^1=yes.

^b^0.5=yes/no.

^c^0=no.

### Characteristics of Included Studies

A total of 10 studies from 6 countries that met the inclusion criteria were included in this study (The Netherlands, n=2; Australia, n=3; Sweden, n=2; Nigeria, n=1; Germany, n=1; and United States of America, n=1; Table 2). These studies were published between 1993 and 2021. The sample size used to estimate the cost-effectiveness of digital health intervention in the included studies ranged from 47 to 779. The included studies were on LBP (n=4), chronic pain (n=3), and knee and hip osteoarthritis (n=3). The economic perspectives adopted in the included studies were societal (n=4), societal and health care (n=3), and health care (n=3); 5 of the included studies performed cost-effectiveness analysis, 2 studies performed cost-utility analysis, and the remaining performed both cost-effectiveness and cost-utility analysis. The time horizon of these studies ranged from 8 weeks to 1 year.

**Table 2:** Characteristics of the included studies.

Author, country	Sample size	Mean age (years)	Follow-up	Methods	Intervention modalities	Comparator	Perspectives	Reference costs	Intervention outcomes
Suman et al [20], The Netherlands	779 (n_intervention_=331 and n_control_=448)	Intervention group=55.7 (SD 13.9); control group=56.6 (SD 14.6)	3 months	RCT^a^/CUA^b^	Access to a multifaceted eHealth strategy: informative website, digital monthly newsletters, and social media platforms	Digital patient information letter; no access to the website, materials, or social media platforms	Societal	Intervention group=€8444 (US $10,000); control group=€8979 (US $10,632.52)	Willingness to pay per quality-adjusted life-year
Dear et al [21], Australia	490 (n_intervention1_=143, n_intervention2_=141, n_intervention3_=131, and n_control_=75)	50 (SD 13)	8 weeks	RCT/CEA^c^	Evidence-based face-to-face pain management programs; participants were also provided with information designed to help people understand their symptoms and difficulties, and to teach cognitive and behavioral self-management skills to help reduce pain-related disability, anxiety, and depression.	Usual care	Health care	N/A^d^	Cost per ≥30% reduction in disability, depression, anxiety, and pain
Ekman et al [30], Sweden	499 (75% female)	65	12 weeks	Literature review/CEA	It consists of a patient interface that provides individually tailored information on osteoarthritis and exercises for rehabilitation and support for lifestyle changes. It includes a provider interface where a trained physiotherapist can follow progress of the patient and provide feedback and support throughout the treatment period.	Face-to-face treatment (two to three 1-hour, physiotherapist-led face-to-face lectures with information about the condition and available treatment)	Societal/health care	Societal costs: intervention group=2776 SEK (US $3234.8); control group=10,610 SEK (US $12,363); health care costs: intervention group=766 SEK (US $892); control group=1299 SEK (US $1513.7)	Cost per unit of effect improvement
Fatoye et al [23], Nigeria	47 (n_intervention_=21 and n_control_=26)	47 (SD 11.6)	8 weeks	RCT/CEA and CUA	Telerehabilitation, a mobile phone–based app of mechanical diagnosis and therapy. Most of these participants were provided with smartphones.	Usual care	Health care	Intervention group=US $61.8; control group=US $106.3	Cost per quality-adjusted life-year
Paganini et al [24], Germany	302 (84.1% female; n=254)	51.7 (SD 13.1)	6 months	RCT/CUA	A guided and unguided internet-based intervention. It consists of 7 modules, which include information, metaphors, assignments, and mindfulness exercises. Participants were advised to work on 1 module per week (~60 minutes).	Usual care and participants were offered to use the unguided internet-based intervention after the last follow-up assessment	Societal	Guided=€6945 (US $8417.8); unguided=€6560 (US $7951.1); control group=€6908 (US $8372.9)	Cost per quality-adjusted life-year
Williams et al [25], Australia	160 (n_intervention_=79 and n_control_=80)	Intervention group=56.0 (SD 13.3); control group=57.4 (SD 13.6)	6 months	RCT/CEA and CUA	An intervention involving brief telephone advice, a clinical consultation with a physiotherapist, and referral to a 6-month telephone-based health coaching service	Usual care (no restrictions were placed on their use of other health services during the study period)	Societal and health care	Intervention group=Aus $1272 (US $1501.3); control group=Aus $1886 (US $2225.9)	Cost per quality-adjusted life-year
Hedman-Lagerlöf et al [26], Sweden	140 (n_intervention_=70 and n_control_=70)	50.3 (SD 10.9)	10 weeks	RCT/CEA	Internet-delivered exposure therapy, comprising about 100 pages divided into 8 modules of self-help material	No treatment	Societal and health care	Intervention group=US $8903; control group=US $11,940	Cost per change on the Fibromyalgia Impact Questionnaire
O’Brien et al [27], Australia	120 (n_intervention_=60 and n_control_=60)	Intervention group=63.0 (SD 11.1); control group=60.2 (SD 13.9)	6 months	RCT/CEA and CUA	Brief advice and education about the benefits of weight loss and physical activity for knee osteoarthritis were provided over the telephone. Participants were informed about the New South Wales Get Healthy Information and Coaching Service and referred to the service for weight loss support.	Usual care	Societal	Intervention group=Aus $4387 (US $5177.7); control group=Aus $3819 (US $4507.3)	Cost per quality-adjusted life-year
Weinberger et al [28], United States	393 (n_intervention_=191 and n_control_=202)	Intervention group=63.34 (SD 10.50); control group=61.1 (SD 12.5)	1 year	RCT/CEA	Participants were called monthly, as well as 1 week prior to scheduled general medicine practice visits.	Received their regular care without telephone intervention	Health care	Intervention group=US $184; control group=US $170	Cost per improvement in physical functioning and pain
de Boer et al [29], The Netherlands	72 (n_intervention_=22 and n_control_=28)	N/A	16 weeks	RCT/CEA	Comprised 8 sessions of 2 hours (7 continuous sessions and 1 booster session 2 months after the last session). Before the start of the course, a manual containing information about how to access the internet course is sent to the participants.	The control group received the same program and content as the intervention group. However, their intervention took place in a meeting room at the hospital and was facilitated by a trained psychologist.	Health care	Intervention group=€1745 (US $2207.2); control group=€1717 (US $2171.8)	Cost per 1 additional point improvement gained on the Pain Catastrophizing Scale

^a^RCT: randomized controlled trial.

^b^CUA: cost-utility analysis.

^c^CEA: cost-effectiveness analysis.

^d^N/A: not available.

### Cost-Effectiveness of Digital Health Interventions

The results of the cost-effectiveness and cost-utility analyses of digital health intervention of the included studies are described in this section (also see Table 3). Except 1 study [27], all the included studies reported that digital health interventions were cost-effective compared with the control group. All the included studies provided the ICER and ICUR indicating the dominance of the interventions.

**Table 3:** Clinical outcomes and incremental cost-effectiveness ratios of the included studies.

Author, country	Clinical outcomes	Incremental cost-effectiveness ratios
Suman et al [20], The Netherlands	Disability score: intervention group=mean 5.1 (SD 4.7); control group=mean 5.9 (SD 5.3) Back pain beliefs score: intervention group=mean 24.7 (SD 6.0); control group=mean 24.8 (SD 6.2)	The incremental cost-effectiveness ratios indicated that the intervention dominated usual care and the probability of cost-effectiveness was 0.85 on a willingness to pay of €10,000^a^/quality-adjusted life-year.
Dear et al [21], Australia	Disability (≥30% improvement): self-guided format=39% (95% CI 30 to 48); optional-guided format=40% (95% CI 32 to 48); clinician-guided format=42% (95% CI 34 to 51); and control group=9% (95% CI 2 to 17) Pain intensity (≥30% improvement): control=8% (95% CI 1 to 15); self-guided format=23% (95% CI 15 to 31); optional-guided format=26% (95% CI 18 to 34); and clinician-guided format=21% (95% CI 14 to 28)	For a ≥30% reduction in disability, depression, anxiety, and pain, the costs were as follows: a self-guided format, Aus $404^b^-Aus $808; an optional-guided format, Aus $314-Aus $541; and the clinician-guided format, Aus $88-Aus $225.
Ekman et al [30], Sweden	N/A^c^	Digital model costs around 25% of the existing face-to-face model of care. Digital model and better management of patients with osteoarthritis report on average a reduction in experienced pain from 5.7 to 3.2 and 5.2 to 4.1 after 12 weeks, respectively. The incremental cost-effectiveness ratio was 8705 SEK^d^/effect improvement.
Fatoye et al [23], Nigeria	Quality-adjusted life-years: intervention group=0.085 (95% CI 0.80 to 0.09); control group=0.084 (95% CI 0.084 to 0.085) Disability: intervention group=15.71 (95% CI 12.85 to 18.57); control group=14.50 (10.63-18.36)	The telerehabilitation was associated with an additional 0.001 quality-adjusted life-years (95% CI 0.001 to 0.002) per participant compared with the clinic-based arm.
Paganini et al [24], Germany	Quality-adjusted life-years (AQoL-8D^e^): ACTonPain_guided_^f^=mean 0.44 (SD 0.05); ACTonPain_unguided_=mean 0.266 (SD 0.09); and waitlist control group=mean 0.244 (SD 0.08) Treatment response (pain interference): ACTonPain_guided_=mean 0.44 (SD 0.05); ACTonPain_unguided_=mean 0.277 (SD 0.04); and waitlist control group=mean 0.158 (SD 0.04)	Treatment response and quality-adjusted life-years were highest in the ACTonPain_guided_ group (44% and 0.280; mean costs=€6945), followed by the ACTonPain_unguided_ group (28% and 0.266; mean costs=€6560) and the control group (16% and 0.244; mean costs=€6908). The ACTonPain_guided_ group versus the control group revealed an incremental cost-effectiveness ratio of €45 and an incremental cost-utility ratio of €604. The ACTonPain_unguided_ group dominated the control group.
Williams et al [25], Australia	N/A	Societal: mean total costs were lower in the intervention group than in the control group (US $614; 95% CI –3133 to 255). Health care: mean total costs were higher in the intervention group than in the control group (US $386; 95% CI –188 to 688). No differences were found between the intervention and control groups in quality-adjusted life-years (mean difference 0.02; 95% CI –0.00 to 0.04), pain (mean difference –0.35; 95% CI –1.33 to 0.64), disability (mean difference –0.57; 95% CI –10.41 to 9.27), weight (mean difference –2.04; 95% CI –4.22 to 0.14), and BMI (mean difference –0.67; 95% CI –1.44 to 0.09). For all outcomes, the intervention was on average less expensive and more effective than usual care, and the probability of the intervention being cost-effective compared with usual care was relatively high (ie, 0.81) at a willingness to pay of US $0/unit of effect.
Hedman-Lagerlöf et al [26], Sweden	EuroQol five-dimensional: intervention group=mean 0.60 (SD 0.30); control group=mean 0.44 (SD 0.32) Fibromyalgia Impact Questionnaire: intervention group=mean 36.44 (SD 25.56); control group=mean 57.51 (SD 21.62)	Intervention group, mean 36.44 (SD 25.56); control group, mean 57.71 (SD 21.62) The mean score on the Fibromyalgia Impact Questionnaire for the experimental group at the 12-month follow-up was 39.95 (SD 21.77), representing a nonsignificant change. The incremental cost-effectiveness ratio was –5025/0.33=–US $15,295. This means that for 1 additional responder to treatment, there was a societal cost saving of US $15,295. Taking a health care unit perspective, the corresponding incremental cost-effectiveness ratio was 726/.33, which equates to around US $2211.
O’Brien et al [27], Australia	N/A	Mean cost differences between groups (intervention minus control) were Aus $493 (95% CI –3513 to 5363) for health care costs, Aus $–32 (95% CI –73 to 13) for medication costs, and Aus $125 (95% CI –151 to 486) for absenteeism costs. The total mean difference in societal costs was Aus $1197 (95% CI –2887 to 6106). For quality-adjusted life-years and all clinical measures of effect, the probability of the intervention being cost-effective compared with usual care was less than 0.36 at all willingness-to-pay values.
Weinberger et al [28], United States	N/A	The costs to achieve 1-unit improvements in physical functioning and pain (US $71.00 and US $31.00, respectively) over 1 year were extremely low. The annual costs for a 1-unit improvement in physical functioning and pain, as measured by the Arthritis Impact Measurement scale, were US $70.86 and US $31.00, respectively.
de Boer et al [29], The Netherlands	Pain Catastrophizing Scale score: intervention group=mean 0.69; control group=mean 0.37 Visual Analog Scale—Pain score: intervention group=mean 0.62; control group=mean 0.06 RAND-36 Physical Functioning score: intervention group=mean 0.42; control group=mean 0.15	Pain Catastrophizing Scale: intervention group, mean 11.00 (SD 11.49); control group, mean 16.10 (SD 11.56) Visual Analog Scale—Pain: intervention group, mean 5.19 (SD 2.53); control group, mean 5.49 (SD 2.32) RAND-36 Physical functioning: intervention group, mean 58.50 (SD 22.37); control group, mean 55.88 (SD 22.35)

^a^€1=US $1.08 (as of June 12, 2023).

^b^Aus $1=US $0.67 (as of June 12, 2023).

^c^N/A: not available.

^d^1 SEK=US $0.09.

^e^AQoL-8D: Assessment of Quality of Life 8-Dimension.

^f^ACT: acceptance and commitment therapy.

### Low Back Pain

A total of 4 studies [20,23-25] reported the cost-effectiveness of digital health intervention on people with LBP. The digital health intervention consisted of an informative website, digital monthly newsletters, and social media platforms [20]; telerehabilitation using a mobile phone–based app [23]; internet-based intervention such as information, metaphors, assignments, and mindfulness exercises [24]; as well as a brief telephone advice, a clinical consultation with a physiotherapist, and referral to a 6-month telephone-based health coaching service [25]. The clinical outcomes measured were back pain beliefs, disability, and health-related quality of life.

All these studies concluded that digital health interventions seem to be cost-effective from the health care and societal perspectives. The multifaceted eHealth intervention that aimed to reduce patients back pain, generated on average, a WTP of €10,000 (US $10,778.53) per QALY at a probability .85 being cost-effective [20]. Telerehabilitation was associated with an additional 0.001 QALY per participant compared with the control condition and the telerehabilitation was dominant [23]. The guided internet-based interventions’ probability of being more cost-effective compared with the control condition increased up to 70% and to 95% at a WTP of €24,415 (US $26,315.78) and €91,000 (US $98,084.62), respectively [24]. For all clinical outcomes, an intervention involving a brief telephone advice, a clinical consultation with a physiotherapist, and referral to a 6-month telephone-based health coaching service was on average less expensive and more effective than usual care, and its probability of being cost-effective compared with usual care was relatively high (ie, 0.81) at a WTP of US $0/unit of effect [25].

**Figure 2:**
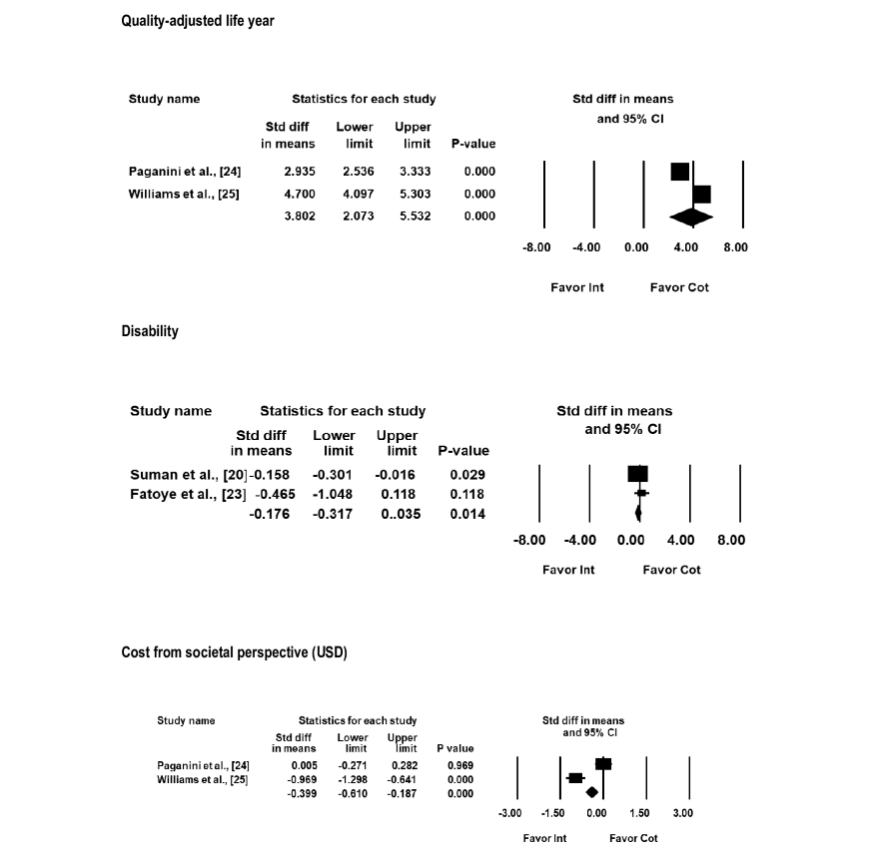
Forest plots of comparison: digital health intervention vs control for low back pain.

A meta-analysis was conducted for disability, QALY, and cost (Figure 2). A statistically significant mean difference in disability (mean difference –0.176; 95% CI –0.317 to –0.035; *P*=.01) and QALY (mean difference 3.855; 95% CI 2.023 to 5.687; *P*<.001) was identified. The meta-analysis for the costs of 2 studies [24,25] favors the digital health intervention compared with the control condition (mean difference –US $0.339; 95% CI –0.610 to –0.187; *P*<.001

### Osteoarthritis

Two studies [27,28] documented the cost-effectiveness of digital health intervention on patients with osteoarthritis. A digital, structured, and individualized treatment program and telephone-based health coaching service were the contents of the digital health interventions. The included studies indicated that patients have improved their health-related quality of life, pain intensity, disability, and physical function due to the interventions. Although 1 of the included studies [27] reported that the digital intervention was not cost-effective, a study conducted in the United States [28] indicated that it is potentially cost-effective in osteoarthritis.

The digital, structured, and individualized treatment program reported on average a reduction in experienced pain from 5.7 to 3.2 (a reduction by 2.5 points on a 0-10 scale, or a 44% reduction) after 12 weeks [30]. Based on the cost-effectiveness ratio, the intervention arm was associated with 8705 SEK (US $805.92) per effect improvement. From the societal and health care perspectives, the mean cost difference between the telephone-based health coaching service and control were US $1197 (95% CI –2887 to 6106) and US $493 (95% CI –3513 to 5363), respectively [27]. For QALYs and all clinical measures of effect, the probability of the telephone-based health coaching service being cost-effective compared with usual care was less than 0.36 at all WTP values. Based on the cost-effectiveness ratio from the health care system perspective, there was an annual cost of US $71.00 and US $31.00 per 1-unit improvement in physical functioning and in pain, as measured by the Arthritis Impact Measurement Scales, respectively [28].

### Chronic Pain

Three studies [21,26,29] compared the cost-effectiveness of digital health intervention with usual care on people with chronic pain. The digital health interventions include therapist-supported treatment comprising about 100 pages divided into 8 modules of self-help material, cognitive-behavioral intervention with email contact of the therapist, and transdiagnostic psychological intervention delivered via the internet. The clinical outcomes were disability, health status (fibromyalgia), physical functioning, and pain. All these studies reported that digital health interventions were cost-effective. For a unit reduction (≥30%) in disability, depression, anxiety, and pain, a self-guided internet-based intervention and control or clinician-guided intervention costs from Aus $404 (US $273.05) to Aus $808 (US $546.09) and Aus $88 (US $59.48) to Aus $225 (US $152.07), respectively [21]. Taking the societal and health care perspectives, the cost-effectiveness ratio between the internet-delivered pain management group and the control group was US $15,295 and US $2211 for 1 additional responder to treatment, respectively [26]. Compared with control, the internet-based cognitive-behavioral intervention showed a saving of €40 (US $43.12) per additional point improvement gained on the Pain Catastrophizing Scale [29].

## Discussion

### Key Findings

To the best of our knowledge, this is the first study to perform a systematic review and meta-analysis on critically appraising the cost-effectiveness of digital health interventions for patients with MSDs. A total of 10 studies from 6 countries were included. The results of our analysis show that there is strong evidence to support that digital health interventions are cost-effective. From a societal perspective, telephone-based weight loss support provided using an existing nondisease-specific 6-month weight management and healthy lifestyle service was not cost-effective in comparison to usual care for patients with osteoarthritis [27]. However, it should be noted that health service delivery systems are not the same in different countries. Moreover, cost-effectiveness results may differ from 1 region to another, as countries have differences in demography and epidemiology.

Nicholl and colleagues [31] conducted a systematic review to critically appraise published evidence concerning the use of interactive digital health interventions to support self-management of LBP. These authors indicated that no information on cost-effectiveness was reported. However, a recently published review [12] has demonstrated that digital health interventions have some clinical benefits in the management of MSDs and may contribute positively toward reducing its multifaceted burden to the individual, economy, and society.

A substantial difference exists in the applicability of digital health interventions in high-income as well as low- and middle-income countries [32]. For example, telerehabilitation, in the form of a mobile phone app platform extension exercise, may be a practical intervention in geographically remote areas [23]. Consistent with our findings, taking a lifetime perspective, the use of a mobile app for the self-management of heart diseases by patients with heart failure (in a Spanish region) appeared cost-effective compared with usual care [33]. Although the type of digital health interventions is one of the influential cost drivers [34], it seems that they are cost-effective in both high- and low-income countries.

Some of the main reasons for the cost-effectiveness of digital health interventions are early identification of a disease using technology and savings made on direct nonmedical costs or indirect costs. Further, prevention of disease progression and its costly health outcomes could be possible through early detection of a disease. For example, those conditions that have been promptly detected or identified could receive intervention or treatment to prevent its progress. Furthermore, using digital health interventions may enable clinicians to reduce time spent for each patient, which in return reduces overall health care resources and in turn burden on the society.

A meta-analysis could not be performed for most of the included studies in this current review. This is because the included studies used different time horizons, different clinical outcomes, and did not present mean or SD for costs. However, it was possible to pool effectiveness and cost data from 2 studies [24,25]. From a societal perspective, compared with the control group, the meta-analysis results of these 2 studies suggest that the digital health intervention is more effective and less costly. This suggests that digital health interventions are cost-effective compared with control. Among other parameters, it is important to consider the transferability of the cost-effectiveness results from other countries before using them in the decision-making process. Therefore, this review could provide useful information that digital health interventions are cost-effective and policy makers can apply regional cost-effectiveness data to their local decision-making.

### Strengths and Limitations

There are certain strength and limitations to this study. In this review, we used a systematic approach such as the screening of numerous databases, the involvement of multiple reviewers, and assessment of methodological quality of the studies. Only English language studies were included. Therefore, it is possible that relevant literature published in other languages may have been excluded. Only a small number of studies met the inclusion criteria. It was also not possible to perform a meta-analysis for most of the included studies due to the adoption of different time horizons and different clinical outcomes; further, many studies did not present mean or SD for costs [35]. Besides, the quality of the included studies indicates that there is a need to interpret the cost-effectiveness results with caution. Finally, we found just 1 study precluding any meaningful conclusions on the cost-effectiveness of digital health interventions. Overall, the findings of this review should be generalized only after taking local information such as perspective, time horizon, currency, and gross domestic product specific for each country into account [36].

### Conclusions

Studies indicate that digital health interventions are cost-effective. Our findings also suggest that digital health interventions could help improve access to treatment for patients with MSDs, thereby improving their health outcomes. Policy makers should consider the use of these interventions for patients with MSDs. This study may provide useful information to decision makers at a time when there is a shortage of economic evidence.

## Appendix

Multimedia Appendix 1 PRISMA (Preferred Reporting Items for Systematic Reviews and Meta-Analyses) checklist.

Multimedia Appendix 2 Search terms.

## Abbreviations

ACT: acceptance and commitment therapy
ICER: incremental cost-effectiveness
ICUR: incremental cost-utility ratio
LBP: low back pain
MSD: musculoskeletal disorder
QALY: quality-adjusted life-year
QHES: Quality of Health Economic Studies
WTP: willingness to pay

## Data Sharing

All data related to this work are available in this research article..
