# Supplementary material for: Economic Evaluations of Digital Health Interventions for the Management of Musculoskeletal Disorders: Systematic Review and Meta-Analysis
Source: J Med Internet Res. 2023 Jul 6;25:e41113. doi: 10.2196/41113 (PMC10359913; doi:10.2196/41113)
Supplement: Multimedia Appendix 2 [file jmir_v25i1e41113_app2.docx]

We searched 7 electronic databases: Medline, AMED, CIHAHL, PsycINFO, Scopus, Web of Science, and Centre for Review and Dissemination for relevant studies. The search strategy in each database is as follows.

**Medline, AMED, CIHAHL, PsycINFO**

S7. S1 AND S5 AND S6

S6. S2 OR S3 OR S4

S5. TI cost-utility or cost utility or cost-benefit or cost benefit or cost-effectiveness or cost

effectiveness or cost consequence or cost-consequence or economic outcome or

economic evaluation or economic impact or health economic* or economic

modelling or economic assessment

S4. TI digital health or mobile health or mhealth or m-health or electronic health or

ehealth or e-health or health technology information

S3. TI Internet or cell phone or telephone or text messaging or electronic mail or mobile

applications

S2. TI telemedicine or remote consultation or telemetry or telenursing or precision

medicine

S1. TI Osteoarthritis Or Rheumatoid Arthritis Or Bone Fractures Or Carpal Tunnel

Syndrome Or Fibromyalgia Or Pain Or Low back pain Or Back pain

**Scopus**

(ALL(Cost-utility or cost utility or cost-benefit or cost benefit or cost-effectiveness or cost effectiveness or cost consequence or cost-consequence or economic outcome or economic evaluation or economic impact or health economic* or economic modelling or economic assessment)) AND (ALL("back pain" Or "neck pain" Or "spininal pain" Or "spin* pain" Or "thoracic pain" Or "cervical pain" Or "lumbar pain" Or "BACK PAIN" OR "LOW BACK PAIN" Or "NECK PAIN" Or "knee pain" Or "shoulder pain" Or "elbow pain" Or ''HAND pain'' Or ''WRIST pain'' OR "ankle pain" Or "foot pain" Or "hip pain" Or "musculoskeletal pain")) AND (ALL(website* OR webpage* Or "web page*" Or "web site*" Or "computer based*" Or "computer assisted*" Or digital Or ''online mobile application app'' OR apps Or internet OR smartphone* OR "smart phone*", digital health or mobile health or mhealth or m-health or electronic health or ehealth or e-health or health technology information, telemedicine or remote consultation or Smart phone, OR internet OR "Mobile application" OR Software or '' software design''))

**Web of Science**

#1 AND #2 AND #3

Query #1

website OR webpage Or web page Or web site Or computer based Or computer assisted Or digital Or online mobile application app OR apps Or internet OR smartphone OR smart phone, digital health or mobile health or mhealth or m-health or electronic health or ehealth or e-health or health technology information, telemedicine or remote consultation or Smart phone, OR internet OR Mobile application OR Software or software design (All Fields)

Query #2

back pain Or neck pain Or spininal pain Or spin pain Or thoracic pain Or cervical pain Or lumbar pain Or BACK PAIN OR LOW BACK PAIN Or NECK PAIN Or knee pain Or shoulder pain Or elbow pain Or HAND pain Or WRIST pain OR ankle pain Or foot pain Or hip pain Or musculoskeletal pain (All Fields)

Query #3

Cost-utility or cost utility or cost-benefit or cost benefit or cost-effectiveness or cost effectiveness or cost consequence or cost-consequence or economic outcome or economic evaluation or economic impact or health economic* or economic modelling or economic assessment (All Fields)
